# Solar-Radiation-Dependent Anisotropic Thermal Management Device with Net Zero Energy from 4D Printing Shape Memory Polymer-Based Composites

**DOI:** 10.3390/ma16103805

**Published:** 2023-05-18

**Authors:** Zhuo Wang, Yao Zhang, Yanhui Niu, Xuejian Chen, Jianrong Song

**Affiliations:** 1 School of Materials Science and Engineering, Chang’an University, Xi’an 710061, Chinaniuyh@chd.edu.cn (Y.N.); 2 State Key Laboratory of Special Functional Waterproof Materials, Beijing Oriental Yuhong Waterproof Technology Co., Ltd., Beijing 101300, China

**Keywords:** 4D printing, shape memory polymers (SMPs), anisotropic thermal conductivity, thermal management

## Abstract

Reports have pointed out that nearly 50% of the global total energy demand for buildings is used for daily heating and cooling. Therefore, it is very important to develop various high-performance thermal management techniques with low energy consumption. In this work, we present an intelligent shape memory polymers (SMPs)-based device with programmable anisotropic thermal conductivity fabricated by a 4D printing technique to assist in thermal management with net zero energy. Highly thermal conductive BN nanosheets were textured in a poly (lactic acid) (PLA) matrix by 3D printing, and the printed composites lamina exhibited significant anisotropic thermal conductivity. The direction of heat flow in devices could be switched programmably, accompanying the light-activated deformation controlled by grayscale of composite, which was demonstrated by the “windows” arrays composed of in-plate thermal conductivity facets and SMPs-based hinge joints, achieving the programmable movement of opening and closing under different light conditions. Based on solar radiation-dependent SMPs coupled with the adjustment of heat flow along anisotropic thermal conductivity, the 4D printed device has been proved in concept for potential applications in thermal management in a building envelop for dynamic climate adaptation, taking place automatically based on the environment.

## 1. Introduction

Buildings account for 30% of global energy consumption and produce 10% of the world’s greenhouse gases. The outdoor ambient temperature of buildings most of the time is constantly changed by factors such as climate and solar radiation, and the distribution of temperature in a building will be different due to forms and internal structures. In total, 48% of building energy consumption is used for daily heating and cooling [1,2,3].

The building envelope is the interface separating a building from external space. Thermal management through a building envelope is mainly achieved by changing the thermal properties of the envelope to reduce the impact of outdoor environmental factors and to save energy, as well as to improve indoor comfort. Kinetic building envelops aiming for dynamic climate adaptation and performance in the built environment and self-responsive formal variation automatically upon external stimulation [4,5] have become a subject of progressive research and development in architecture [6,7]. The key factor to realize thermal management is the device having the ability to regulate the heat flow of the vertical building interface by shading the radiation of sunlight or switching the dual-mode radiative thermal management between solar heating and radiative cooling with temperature based on SMEs by changing its own physical-chemical properties [8,9]. Tolley et al. [10] presented self-foldable paper laminates that could be spontaneously driven by the thermal activation of a shape-memory polymer (SMP) deployed in between as a hinge joint. Wu et al. [11] demonstrated that the programmed composition of mechanically different SMPs enabled the evolution of curling shapes exhibiting multiple shape memory effects (SMEs). Zhang [12] furthered these approaches to achieve tunable thermal radiative properties according to varying cooling requirements by the deformation of SMP composites responding to ambient temperature. Based on the mechanism of SMPs deformation, sensor-less, flexible, lightweight, and zero energy building mobility is enabled with reduced building envelop complexity and energy consumption [8,13].

The additive manufacturing technology of 4D printing shape memory polymers (SMPs)-based composite materials is derived from the traditional 3D printing process for intelligent materials and intelligent structure [14]. The shape changing properties of 3D printing SMPs structures are triggered by external stimuli including temperature, solvent, pH value, etc. The realization of 4D printing requires the effective cooperation of intelligent structures, external driving mechanisms and an intelligent material additive manufacturing process [15,16,17,18].

Nowadays, most of the reports on adaptive devices for thermal management are concentrated on the heat flow in a vertical direction on the building interface [9,12,13,19]. Concerning the nature of the heat conduction process governed by Fouries’s law, heat flow is directly proportional to the thermal conductivity of the heat conductor and inversely proportional to the gradient of the temperature. The radiation originated from sunlight not only generates heat flow vertically, but also drives it parallel to the building interface; the latter could balance the temperature variation in different parts of buildings to improve indoor comfort. Therefore, associated with the shape memory behavior of SMPs, a promising method of regulating the direction of heat flow based on the anisotropic thermal conductivity of a polymer matrix filled with 2D fillers (h-BN, graphene, MXenes, etc.) [20,21,22], the direction-shifting of thermal conductivity could be coupled with the deformation of shape or structure constructed by SMPs with anisotropic physical properties. Originating from such concept, we developed an intelligent shape memory polymers (SMPs)-based device with programmable anisotropic thermal conductivity fabricated by 4D printing as a building envelope for thermal management. *h*-BN nanosheets were aligned in the printing nozzle, and printed composites demonstrated anisotropic thermal conductivity. The direction of heat flow in the textured composites could be switched, which was coupled with the sunlight-triggered programmable deformation spontaneously via temperature-sensitive grayscale. Such programmable movement based on shape memory behavior has been proved in the concept of potential applications for thermal management in building envelopes for dynamic climate adaptation, taking place automatically based upon the environment.

## 2. Materials and Methods

### 2.1. Materials

Boron nitride (*h*-BN) (GR, 99%) with an average size of 22 μm was purchased from Hebei Metallurgical Powder Research Institute. Dichloromethane (CH_2_Cl_2_, AR, 99%) was purchased from Hailian Chemical Glass & Instrument Chemical Co., Ltd., Xi’an, China. Graphene (GR, 99.9%) with average size of 0.65 μm was provided by Xingsheng Rare Metal Company, Xingtai, China. Poly (lactic acid) (PLA) pellets (Mw = 150,000) were provided by Hubei Hanlong Bio-Tech Co., Wuhan, China. All the materials were used with no further treatment.

### 2.2. Modification of Fillers

A 3.0 g quantity of bulk *h*-BN powder was boiled in aqueous 5.0 mol/L NaOH at 120 °C for 24 h. The product was washed and centrifuged at 2000 rpm to remove residual NaOH, and then dispersed in 400 mL of deionized water and sonicated at 30 °C for 10 h. The aggregates were removed by centrifugation at 3000 rpm to obtain hydroxylated BN nanosheet (OH-BNNS) aqueous dispersions. The OH-BNNS was collected after the DI water was removed.

### 2.3. Preparation of Composite Lamina

For the preparation of the composite lamina, 2 g PLA and h-BN, OH-BNNS with 5% to 25% mass ratio of PLA were weighed and mixed with 25 mL of dichloromethane (CH_2_Cl_2_). Then, 2 g PLA and graphene with 1%, 2%, 3%, 4% and 5% mass ratio of 20 wt% of *h*-BN were weighed and mixed with 25 mL of dichloromethane (CH_2_Cl_2_). The mixture was magnetic stirred for two hours.

The laminas with different thickness were tape-casted onto a clean glass substrate to fabricate uniform wet films, which were placed on an 80 °C heating plate immediately to rapidly evaporate solvent. After heating to 80 °C to remove the residual solvent, the SMPs-based tape was easily removed from the substrate. The tape film can be tailored to the desired shape and further fabricated into thermal management products.

The solvent in homogeneous slurries was removed in a rotatory evaporator at 80 °C, and the obtained dry composites were shredded into granules for 3D printing. The lamina was performed on a desktop 3D printer (Model G5, Shenzhen Creality 3D Technology Co., Ltd., Shenzhen, China) with the capability for programmable patterning in three dimensions. The printer’s mechanical arm featured a maximum resolution of 5 μm per axis. A nozzle with an inner diameter of 800 μm was used and granules were loaded in the screw extrusion system. The printing process was achieved at 210 °C and printing speed was fixed at 30 cm/min. The laminas with different filler-loading were printed on tempered-glass substrate at 45 °C. Generally, the in-plate strain in multi-ply lamina is regulated by the printing pattern [23]. To avoid shape-shifting in self-bending made by a longitudinal shrinking top layer and self-twisting made by changing the orientation of filament in the top layer, the direction of parallel filaments in each printed layer was at an angle of 45° to the edge of the lamina and perpendicular to adjacent layers and all printed layers were stacked to fabricate multi-ply lamina.

### 2.4. Assembly of “Windows” Array

We designed the “windows” arrays composed of printed facets with 50 mm × 50 mm × 0.5 mm with granules of BNNS/PLA and a hinge joint of 30 mm × 10 mm × 0.2 mm with granules of graphene/OH-BNNS/PLA. The modes of prototype were designed by AutoCAD2021 (Autodesk Co., San Francisco, CA, USA) and files in “.stl” form were inputted to a 3D printer.

The whole arrays, including frames (pure PLA), were fabricated by 3D printer. The “window” arrays had two modes of shape recovery, in “Open” and “Close” depending on the initial fixed shape of the hinge joint, in programmable steps. The as-prepared lamina filled with graphene was bent at 120 °C (programming temperature) and locked by quenching in ice-water mixture to finish the programming treatment.

### 2.5. Characterization

The powder XRD pattern was measured using a D8 Advance (Bruker AXS, Fitchburg, WI, USA) with a Cu Kα radiation source. The microstructure was characterized by scanning electron microscopy (S4800, Hitachi, Japan). The Shimadzu Fourier Transform Infrared Spectrometer (IRAffinity-1S FTIR) was used to obtain the infrared spectrum of transmittance of the prepared samples. IR images of sample were recorded with a visual IR thermometer (VT04, FLUKE, Everett, WA, USA). The thermal conductivity of printed lamina was measured by laser flash analyzer (LFA467, NETZSCH, Selb, German) at 30 °C.

## 3. Results and Discussion

### 3.1. Material Characteristics

**Figure 1:**
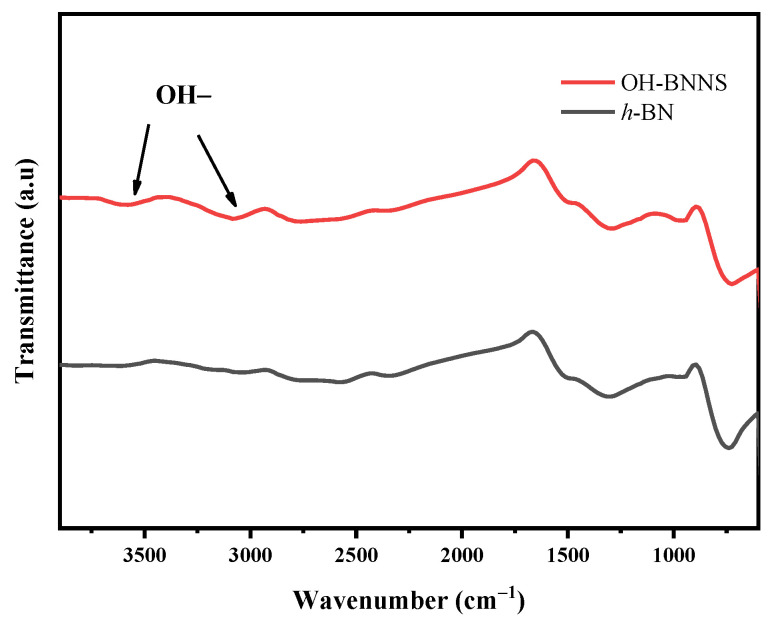
FTIR of *h*-BN and NaOH treated BN samples.

Figure 1 shows a typical infrared spectrum of transmittance of *h*-BN of the in-plane B-N stretching vibrations at 1372 cm^−1^ and out-of-plane B-N bending at 814 cm^−1^. As a comparison, vibrations at 2200 cm^−1^ and 3650 cm^−1^ confirmed the presence of hydroxyl groups in the BNNS after NaOH treatment of *h*-BN. Different from the hydrophobic property of *h*-BN, OH groups in BNNS improved the wettability and benefitted the homogeneous distribution of filler in the PLA matrix.

### 3.2. Textured h-BN, OH-BNNS and Graphene in Composite Lamina

**Figure 2:**
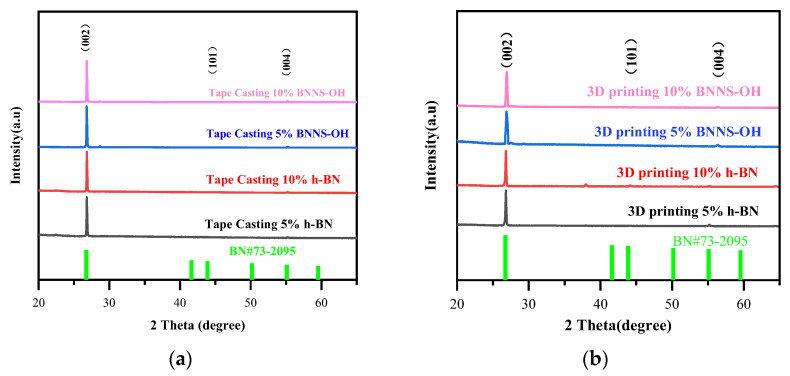
XRD spectrum of samples fabricated by (**a**) tape-casting and (**b**) 3D printing.

**Figure 3:**
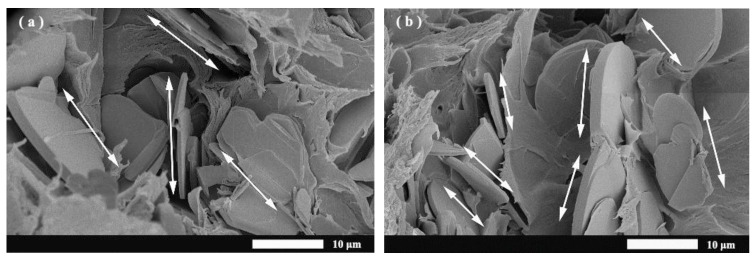

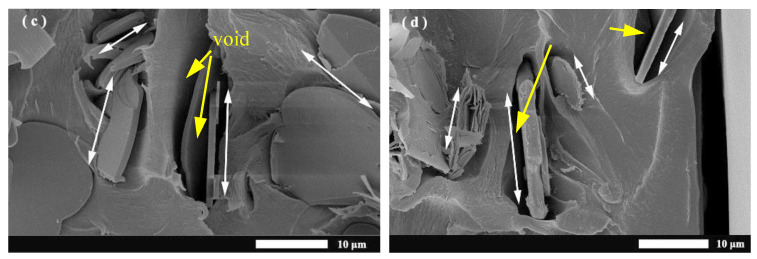
SEM images of (**a**) tape-casting OH-BNNS/PLA composite, (**b**) 3D printing OH-BNNS/PLA composite, (**c**) tape-casting BN/PLA composite and (**d**) 3D printing BN/PLA composite (white arrows are the directions of aligned fillers).

As shown in Figure 2, the powder X-ray diffraction (XRD) pattern of samples of tape-casting (Figure 2a) and 3D printing (Figure 2b) indicates a typical hexagonal crystal of *h*-BN, with a strong and sharp diffraction peak at 2θ = 27° (corresponding to the (002) plane) confirming the 2D nature of the BN nanosheets. The shear-stress generated from the blade in tape-casting helps to align the 2D nanosheets along the thick film, and the alignment of 2D BN nanosheet in the 3D-printed structure is based on the same mechanism. Compared with the tape-casting technique, the shear stress in the 3D printer nozzle could align the 2D nanosheet of *h*-BN and OH-BNNS more effectively, indicated by the ratio of intensity (I_(002)_/I_(101)_), as shown in Table 1. The high length-to-thickness ratio of the 2D BN nanosheets should contribute to a high anisotropy factor in the thermal conductivity of the in-plane and cross-plane directions, and therefore a hierarchically textured BN structure in the composite lamina fabricated by 3D printing with massively continuous and interconnected heat-conduction pathways. Deriving from the force between the OH group on the surface of OH-BNNS and molecule of PLA could contribute to the orientation of the nanosheet in the matrix by the shear stress in extrusion-based 3D printing.

### 3.3. Microstructure of Samples

The scanning electron microscopy provided more information about the alignment of *h*-BN and OH-BNNS in the matrix by tape-casting and 3D printing process. Figure 3 illustrates the SEM images of the cross-section appearance. The shear stress generated in tape-casting and the extrusion of the filament during parallel printing helps the 2D BN and BNNS filler orient along the textured printed lamina. The orientation of the 2D filler in the printed composite is more consistent than that in the casted ones. The direction of alignment is indicated by double arrows in SEM images. Accordingly, micro-level voids (yellow arrow) near the BN sheet in Figure 3c,d were generated from the hydrophobic property of BN on PLA. As a contrast, the samples of OH-BNNS embedded in the PLA matrix without obvious void around fillers are shown in Figure 3a,b, fabricated by tape-casting and 3D printing, respectively, which was attributed to the OH group on the surface of BNNS to modify the wettability verified by FTIR spectrum.

### 3.4. Anisotropic Thermal Conductivity

The hierarchically textured BN or OH-BNNS in composite lamina, featuring in the printed filament at the macroscale with parallel aligned BN and parallel aligned BN nanosheets at the microscale, is highly promising for thermal management. To explore this application, we investigated the thermal properties of the parallel aligned BN in the printed lamina. Adding the SMPs matrix could endow the textured-BN with good flexibility and shape memory propertied for real applications as a programmable thermal management material in kinetic building envelopes. We also prepared samples composed of textured BN/OH-BNNS nanosheets using tape casting as controls.

**Figure 4:**
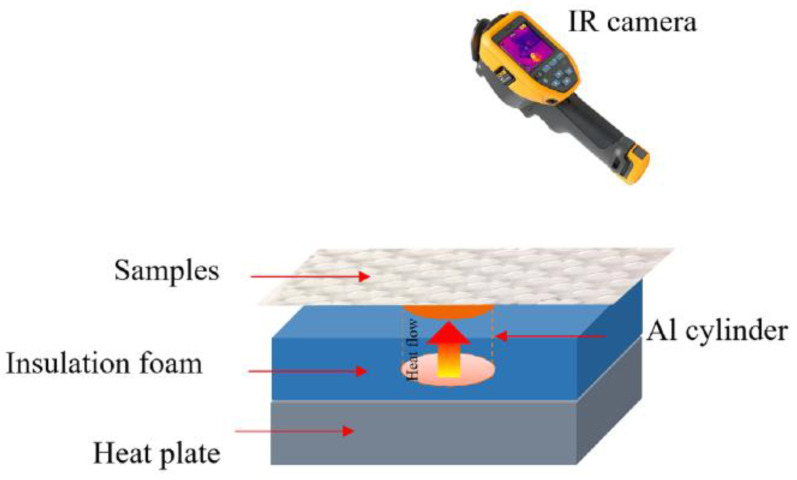
Graphical illustration of the hotspot-IR camera thermal conductivity measurement system.

**Figure 5:**
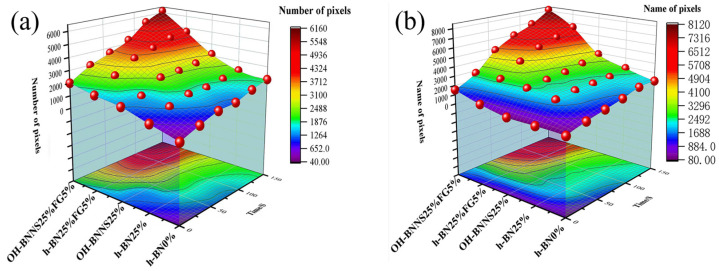
Pixel number colormap of IR images of (**a**) tape-casted and (**b**) 3D-printed laminas.

In the textured-BN/PLA laminas, we expected that the parallel-aligned BN fillers would serve as heat conduction pathways in the plate, thereby effectively removing heat away from the hot source. We evaluated the in-plate thermal conductivity of the textured-BN/PLA and control samples on a homemade hotspot-IR camera system, as shown in Figure 4, using a dynamic-state method. During the test, a hot plate with 100 °C was applied at the bottom of an Al cylinder embedded in insulation foam, and the heat was conducted to the surface of the sample through the Al cylinder. The heat conduction process is governed by Fouries’s law, which states that the heat flow is directly proportional to the thermal conductivity of the heat conductor and inversely proportional to the gradient of the temperature. In this case, a board temperature distribution was attributed to the efficient dissipation of heat when the contact area of samples with the Al cylinder were the same. We evaluated the in-plate thermal conductivity of BN/PLA lamina using the pixel of IR images as the function of time and variation of samples, summarized in Table 2 and Table 3. The 3D colormap shows that the pixel integrals in same sample increased as a function of time, representing the dissipation of heat parallel to the sample surface based on Fouries’s law. The number of pixels of samples in Figure 5b for 3D printing were higher than those of the corresponding ones using tape-casting (Figure 5a) during the whole testing time of 150 s, which illustrates that 3D printing is more efficient than tape-casting in the alignment of 2D filler in textured composites. Such a result was proved by the adding 2D fillers of BN, OH-BNNS and graphene; all these anisotropic thermal conductive fillers could contribute in-plate thermal conductivity with an aligned and textured matrix. A benefit from the interaction of the OH-group in BNNS with PLA matrix was the distribution of OH-BNNS being more homogeneous than *h*-BN, which will lead to higher in-plate thermal conductivity. The addition of graphene not only significantly increased the thermal conductivity, but it also acted as a pigment to adjust the grayscale of the *h*-BN/PLA composite, responding to solar radiation by light absorption with a variation in loadings. The temperature of the samples adjusted by the sunlight absorption could trigger the shape memory behavior of the PLA composite, and the variation in temperature could achieve programmable deformation in sequence as the function of graphene loadings.

**Figure 6:**
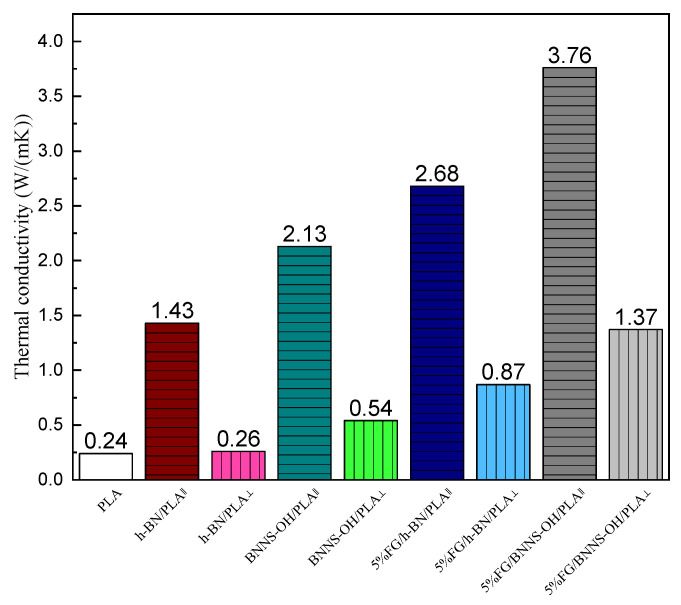
The thermal conductivity of printed lamina with BN or OH-BNNS loading of 20 wt% in-plate (||) and cross-plate (⊥).

The anisotropic thermal conductivity of the printed lamina in-plate and cross-plate is illustrated in Figure 6. The printed laminas exhibit higher in-plate thermal conductivity than cross-plate, which indicates that there was more heat flow parallel than perpendicular to the textured surface with the aligned 2D filler defined as anisotropic thermal conductivity. As shown in the SEM image of Figure 3c,d, the voids near the surface of the BN filler increased the phonon scattering and led to higher thermal resistance, which could be repaired microscopically through the enhancement of the hydrophilic property of OH-BNNS to the PLA matrix.

### 3.5. Photothermal Behavior

**Figure 7:**
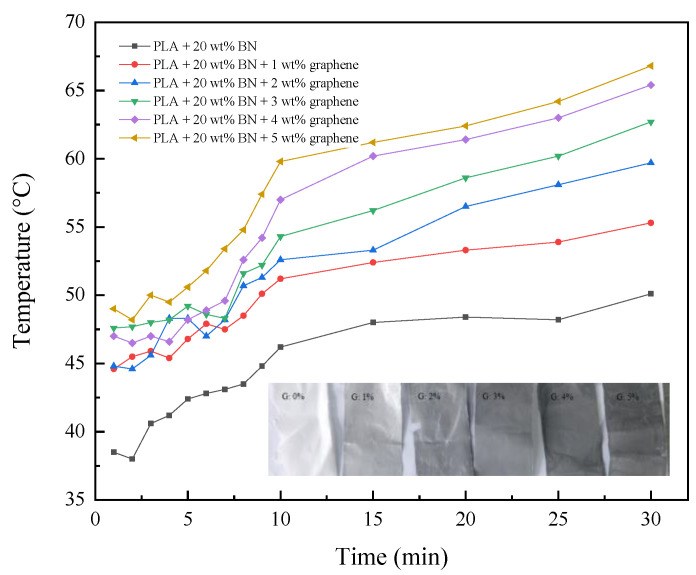
The surface temperature of lamina as a function of graphene loading.

The photothermal effect is a phenomenon which produces thermal energy from electromagnetic radiation. Part of the vibrational energy is transformed into kinetic energy of the electrons, and then converted into heat energy through the relaxation process between electrons and lattices. The SMPs composites exposed to light radiation can perform shape recovery due to the photothermal effect. The photothermal effect of graphene-filled PLA matrix has been investigated in an ambient environment. The test point was located at 108° E and 34° N, with temperature of 32 °C at 10 a.m. The surface temperature of the samples was recorded with an IR camera, and the variation of temperature as a function of time is illustrated in Figure 7. The images of samples with increasing grayscale according to graphene loading from 1 wt% to 5 wt% of 20 wt% *h*-BN-filled PLA matrix are shown in the inset of Figure 7.

In the first 10 min, the surface temperature of all the samples raised dramatically, and in the subsequent time the temperature increased slowly for the thermal equilibrium which was achieved when the heat from sunlight and dissipation to the ambient atmosphere by radiation was equal. Obviously, the addition of graphene to the BN/PLA composite could help the absorption of solar radiation. The surface temperatures of samples depended on the loadings of the graphene filler in the PLA matrix. Therefore, the photothermal behavior originating from radiation absorption could be applied in the programmable movement of intelligent SMPs-based devices as the function of grayscale for the dynamic climate adaptation of buildings happening automatically with respect to the environment.

### 3.6. Programmable Thermal Management by Shape Memory Behavior

**Figure 8:**
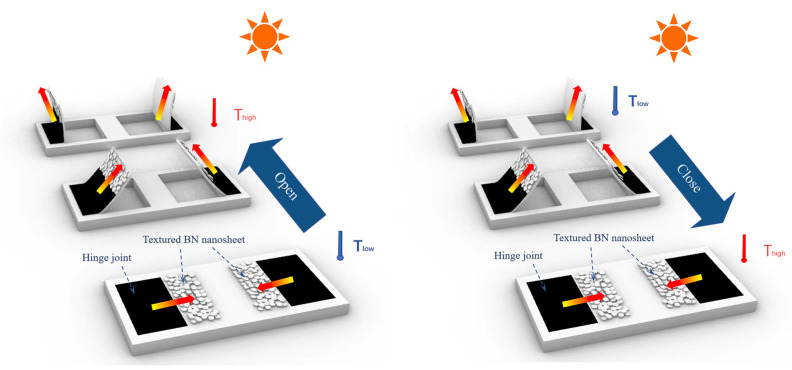
Graphical illustration of programmable thermal management device.

Figure 8 illustrates a prototype of a programmable thermal management device with a textured BN nanosheet facet and light-activated hinge joint fabricated by 3D printing. The device in the form of a “window” had two modes of shape recovery in “Open” and “Close” positions depending on the initial fixed shape in programmable steps at 120 °C. The temperature of the dark hinge joint increased while exposed to the light, and the “window” could determine the movement to open or close. Synchronously, the direction of the in-plate heat flow in the printed, textured BN/PLA facet was shifted from parallel to perpendicular to the window frame.

**Figure 9:**
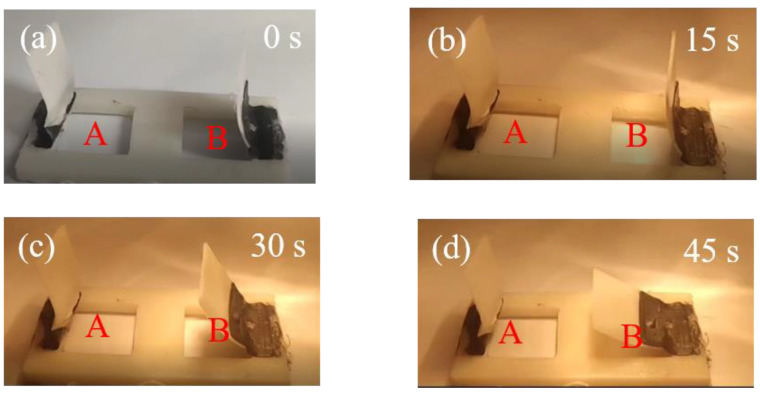
Programmable movements of windows in “Open” mode (A and B in red are labels of windows).

In view of this concept, we constructed two sets of windows made entirely with 3D printing. As shown in Figure 9, the set was composed of two windows in close mode. To simulate the clouds or building shadows, we blocked the light radiation on “window A”. The programmable hinge joint of “window B” recovered from bending to a flat shape, while there was no shape recovery of “window A” within 45 s. The programmable target and results of this set are summarized in Table 4.

The results show that once the light radiation was blocked, the shape memory polymer composites could not be heated above their T_g_ by the photothermal effect and SMPs could not perform shape recovery.

**Figure 10:**
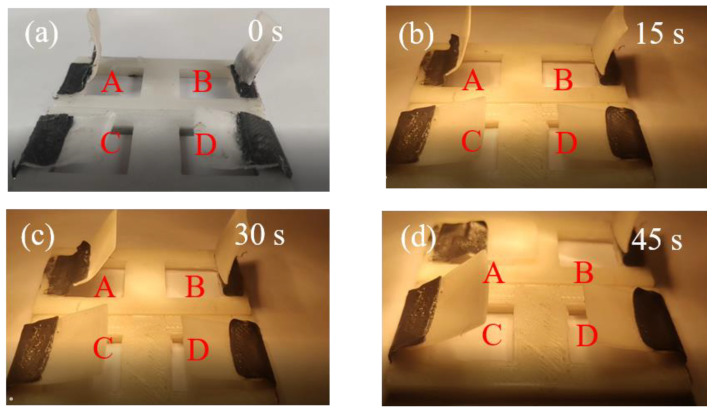
Programmable movements of window arrays in “Open” and “Close” modes (A~D in red are labels of windows).

As shown in Figure 10, both modes of “Open” and “Close” were integrated in a set of devices and we investigated the programmable thermal management in light-on (sunny) and light-blocked (cloudy or shadow) conditions. The results summarized in Table 5 proved in concept that the arrays of “windows” were driven programmably by shape memory behavior, and this could be a potential thermal management device for dynamic climate adaptation and self-responsive shape variation automatically upon environmental stimulation. As for the fused deposition modeling (FDM) in our study, the stretching and alignment of polymer chains of PLA along the direction of extrusion was stored in composite as memory during the printing process, and therefore the shape memory behavior of printed PLA-based composites could be promoted and achieve programmable movements from different initial memory stages [23]. Compared with reports [8,19] on dynamic solar shading devices stimulated by electrical current, our prototype device, driven by solar radiation, has energy-consuming advantages. Furthermore, the regulation of heat flow in 3D printed SMPs structures based on anisotropic thermal conductivity not only works for solar shading by deformation, but could also achieve thermal management both in vertical and parallel directions of the building skin.

## 4. Conclusions

In this study, we introduced OH-groups into the BNNS to improve the wettability of PLA to the surface of fillers, which could benefit the homogeneous distribution of fillers and contribute to the orientation of nanosheets in a textured matrix by the shear stress in extrusion-based 3D printing. We demonstrated the alignment of fillers in a printing process by I_(002)_/I_(101)_ in X-Ray diffraction and anisotropic thermal conductivity along the direction of textured fillers in printed multi-ply laminas. The photothermal behavior originating from radiation absorption could be applied in programmable movements of intelligent SMPs-based devices as the function of grayscale, and the direction of heat flow in textured composites could be switched spontaneously. In view of such a concept, we successfully illustrated a prototype of a programmable thermal management device with two modes of shape recovery composed of a textured BN nanosheet facet and a light-activated hinge joint fabricated by 3D printing, which could be a potential thermal management device aimed at dynamic climate adaptation and automatic self-responsive shape variation in response to environmental stimulation.

## Figures and Tables

**Table 1:** Intensity of the diffraction peaks and intensity ratio of I_(002)_/I_(101)_.

Crystalline Surface	(002)	(101)	(004)	I_(002)_/I_(101)_
PDF card (P-6m2 (187))	67%	15.3%	4.0%	4.38
Tape Casting 5% *h*-BN	91.3%	2.3%	4.1%	22.27
Tape Casting 10% *h*-BN	93.1%	1.2%	3.1%	30.03
Tape Casting 5% OH-BNNS	96.2%	0.4%	2.7%	35.62
Tape Casting 10% OH-BNNS	97.5%	0.5%	1.9%	51.32
3D printing 5% *h*-BN	93.1%	1.1%	3.2%	29.09
3D printing 10% *h*-BN	95.2%	0.9%	2.4%	39.67
3D printing 5% OH-BNNS	96.1%	0.4%	1.9%	50.57
3D printing 10% OH-BNNS	97.5%	0.2%	1.2%	81.25

**Table 2:** Result of hotspot-IR thermal conductivity measurement of tape-casted laminas.

	Time	0 s	30 s	60 s	90 s	120 s	150 s
Tape-Casting							
PLA		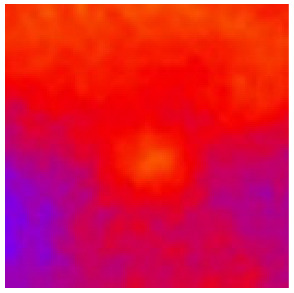	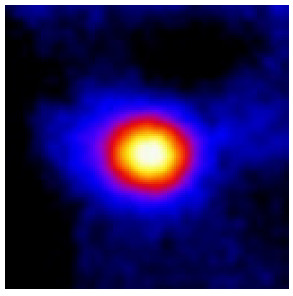	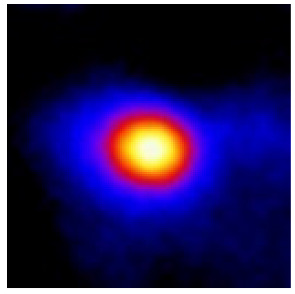	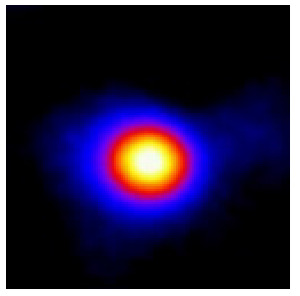	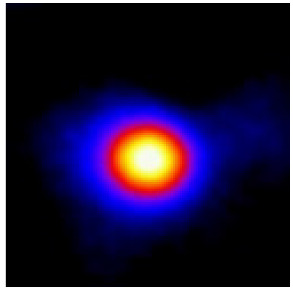	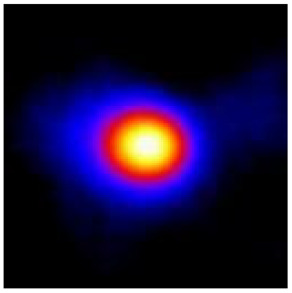
*h*-BN (20%)		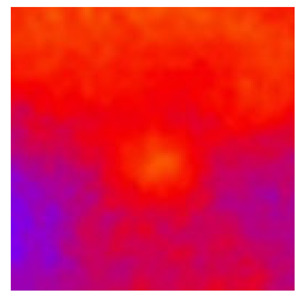	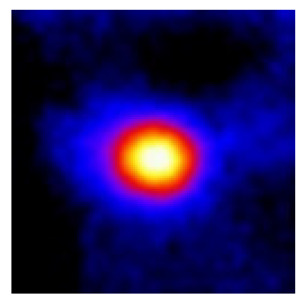	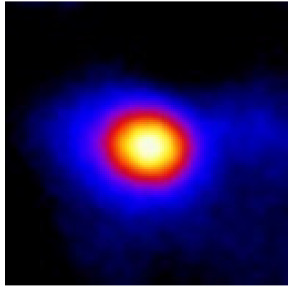	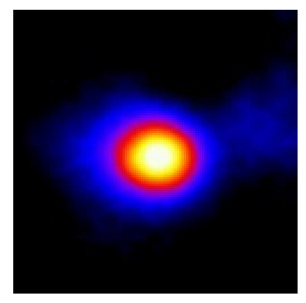	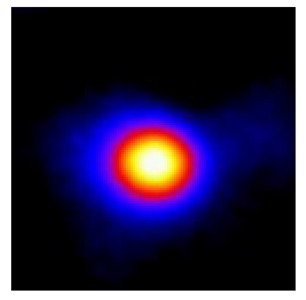	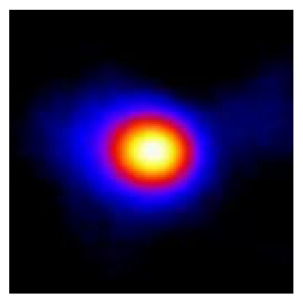
OH-BNNS (20%)		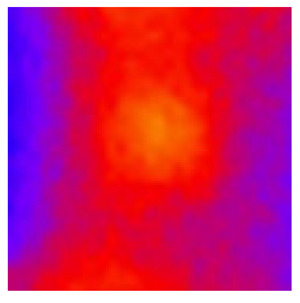	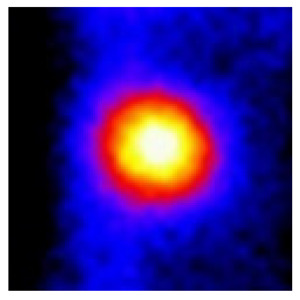	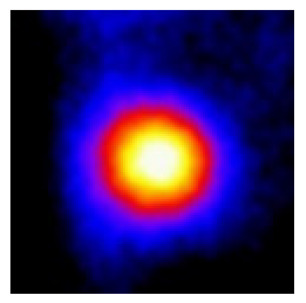	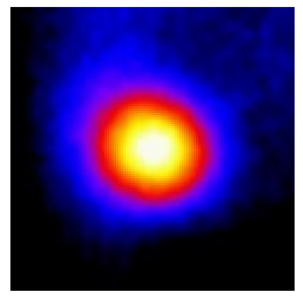	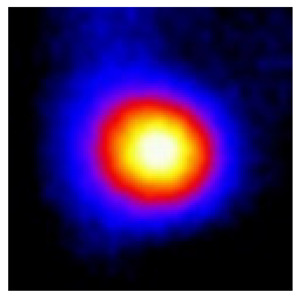	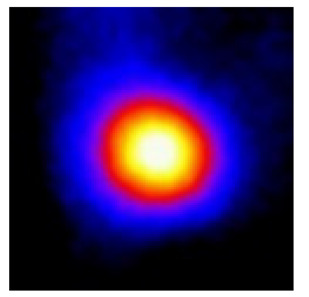
OH-BNNS (20%) + Graphene (5%)		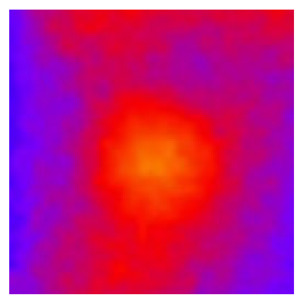	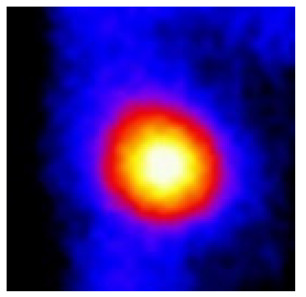	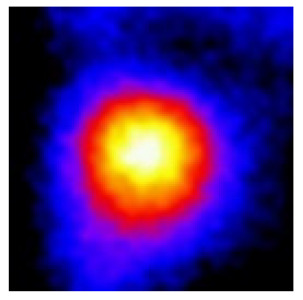	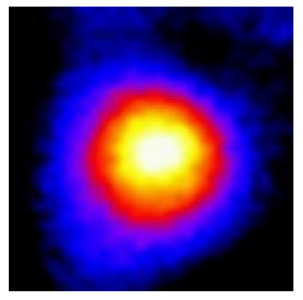	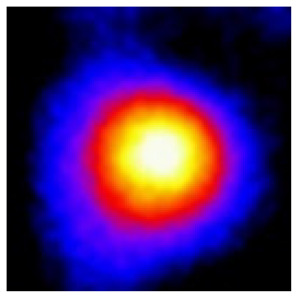	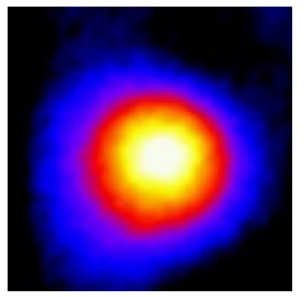

**Table 3:** Result of hotspot-IR thermal conductivity measurement of 3D-printed laminas.

	Time	0 s	30 s	60 s	90 s	120 s	150 s
3D Printing							
PLA		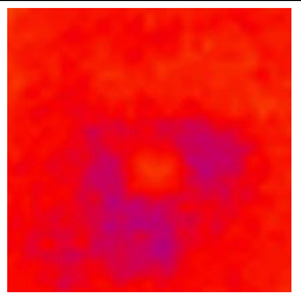	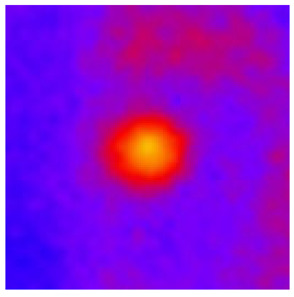	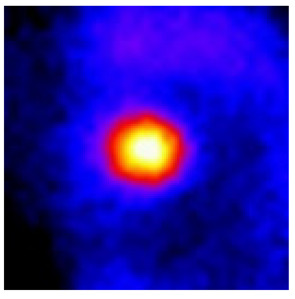	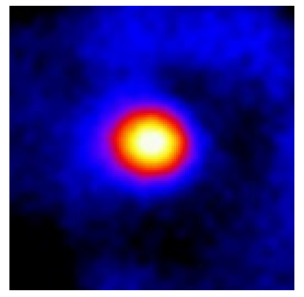	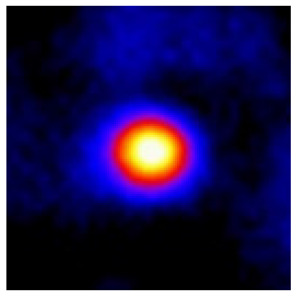	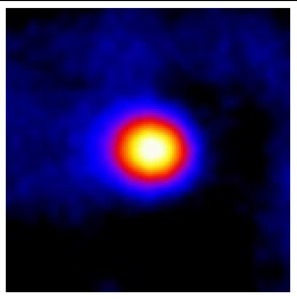
*h*-BN (20%)		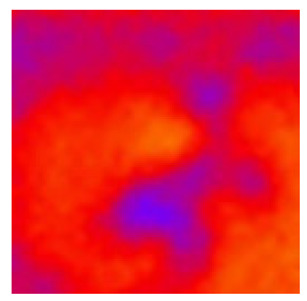	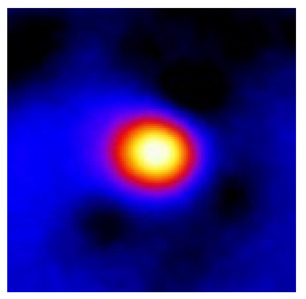	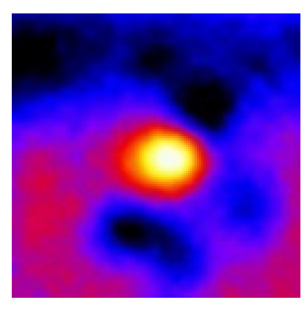	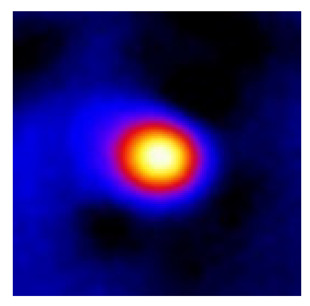	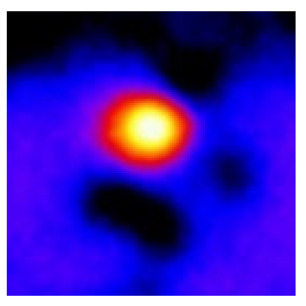	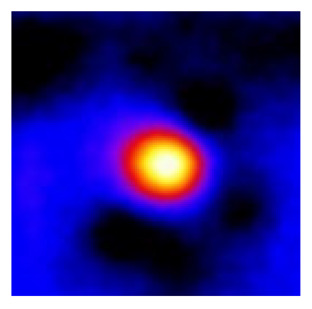
OH-BNNS (20%)		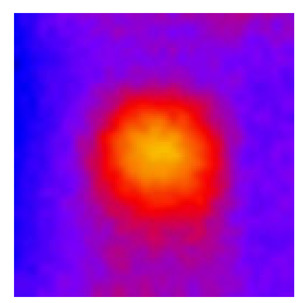	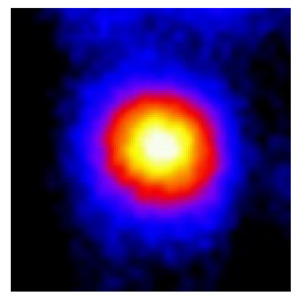	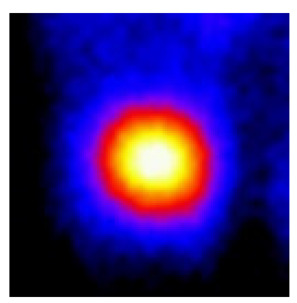	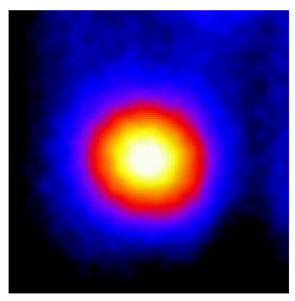	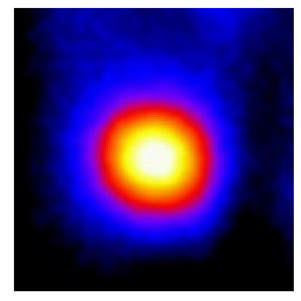	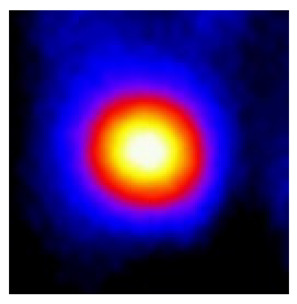
OH-BNNS (20%) + Graphene (5%)		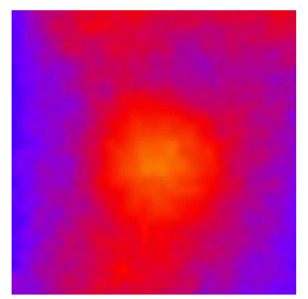	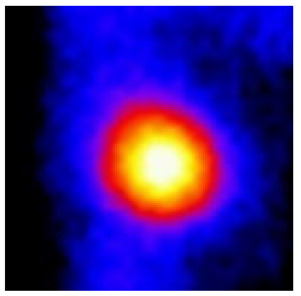	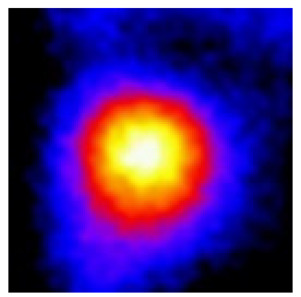	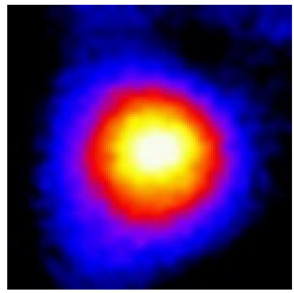	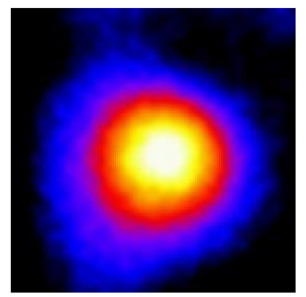	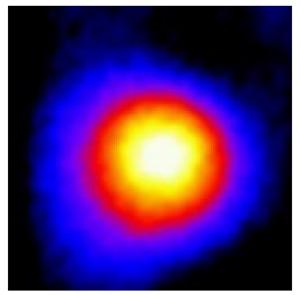

**Table 4:** Summary of programmable movements and responses to light conditions in “Open” mode. (“+” and “0” stand for the condition being applied and not, respectively).

	Light	Shadow	Programmable Target	Original	Results
Window A	+	+	close	open	open
Window B	+	0	close	open	close

**Table 5:** Summary of programmable window movements and responses to light conditions both in “Open” and “Close” modes (“+” and “0” stand for the condition being applied and not, respectively).

	Light	Shadow	Programmable Target	Original	Results
Window A	+	0	close	open	close
Window B	+	+	close	open	open
Window C	+	0	open	close	open
Window D	+	+	open	close	close

## Data Availability

Data sharing not applicable.
